# Understanding the Interfacial Behavior of Cycloaliphatic-like Epoxy Resin with Optical Fibers: Insights from Experiments and Molecular Simulations

**DOI:** 10.3390/ma18163830

**Published:** 2025-08-15

**Authors:** Jianbing Fu, Zhifan Lin, Junhao Luo, Yufan Zheng, Yuhao Liu, Bin Cao, Fanghui Yin, Liming Wang

**Affiliations:** 1 Tsinghua Shenzhen International Graduate School, Tsinghua University, Shenzhen 518055, China; 2 China Electric Power Equipment and Technology Co., Ltd., Beijing 100052, China; 3 College of Electrical Engineering and Automation, Fuzhou University, Fuzhou 350108, China

**Keywords:** optical fiber, cycloaliphatic-like epoxy resin, interface, structure–property relationship

## Abstract

Optical fiber composite insulators are essential for photoelectric current measurement, yet insulation failure at embedded optical fiber interfaces remains a major challenge to long-term stability. This study proposes a strategy to replace conventional silicone rubber with cycloaliphatic-like epoxy resin (CEP) as the shed-sheathing material. Three optical fibers with distinct outer coatings, ethylene-tetrafluoroethylene copolymer (ETFE), thermoplastic polyester elastomer (TPEE), and epoxy acrylate resin (EA), were evaluated for their interfacial compatibility with CEP. ETFE, with low surface energy and weak polarity, exhibited poor wettability with CEP, resulting in an interfacial tensile strength of 0 MPa, pronounced dye penetration, and rapid electrical tree propagation. Its average interfacial breakdown voltage was only 8 kV, and the interfacial leakage current reached 35 μA after hygrothermal aging. In contrast, TPEE exhibited high surface energy and strong polarity, enabling strong bonding with CEP, yielding an average interfacial tensile strength of approximately 46 MPa. Such a strong interface effectively suppressed electrical tree growth, increased the average interfacial breakdown voltage to 27 kV, and maintained the interfacial leakage current below 5 μA even after hygrothermal aging. EA exhibited moderate interfacial performance. Mechanism analysis revealed that polar ester and ether groups in TPEE enhanced interfacial electrostatic interactions, restricted the mobility of CEP molecular chain segments, and increased charge traps. These synergistic effects suppressed interfacial charge transport and improved insulation strength. This work offers valuable insight into structure–property relationships at fiber–resin interfaces and provides a useful reference for the design of composite insulation materials.

## 1. Introduction

With the rapid advancement of new power systems, photoelectric current measurement devices are increasingly replacing traditional electromagnetic equipment due to their superior sampling accuracy, fast response, and strong electromagnetic interference immunity [1,2,3,4]. These devices are now widely deployed at key grid nodes, such as converter stations [5]. Optical fiber composite insulator, as a crucial component of the photoelectric current measurement device, not only facilitates signal transmission but also provides electrical insulation and structural support, both of which are essential for the stable operation of the measurement system [6,7]. The optical fiber composite insulator typically consists of an outer silicone rubber skirt, a fiberglass-reinforced core rod, and embedded optical fibers. One structure involves threading the optical fiber through a hollow core rod and sealing it with filler materials. However, this structure often fails to establish a stable interface between the filler material and the optical fiber, leading to tiny defects such as air gaps and water vapor channels, which may cause partial discharge or even insulation failure. Another structure directly embeds the optical fiber between the core rod and the silicone rubber skirt. Although silicone rubber exhibits excellent hydrophobicity, weather resistance, and flexibility, its inherent non-polar molecular structure makes it difficult to form a strong bond with the optical fiber surface [8,9,10,11]. In practical operation, the interface between optical fiber and silicone rubber skirt is prone to delamination and discharge under the combined effects of high electric fields, high humidity, high temperatures, and mechanical loads. Currently, failures of optical fiber composite insulators due to interface failure are frequent, becoming a weak link that affects the long-term operational reliability of these insulators [11,12,13,14,15].

At present, some preliminary studies have been carried out on the interface failure issue in optical fiber composite insulators. Fault analysis has found that interface failures are primarily concentrated at the high- and low-voltage ends, with visible discharge traces in the optical fiber channels. Simulation studies show that the electric field intensity at the high- and low-voltage ends is significantly higher than at other locations. At high electric field strengths, the interface between different materials tends to accumulate space charges, inducing field distortion and partial discharge, ultimately leading to breakdown along the optical fiber channels. Reducing the electric field strength at the high- and low-voltage ends can moderately enhance the long-term stability of the optical fiber composite insulator [16,17]. Moreover, interfacial failure of optical fiber composite insulators is more pronounced in humid environments. The silicone rubber skirt exhibits high permeability to water and moisture [18]. When combined with defects such as poor sealing at the insulator ends, this allows water vapor and acidic substances to penetrate and migrate along the optical fiber interface. This penetration can trigger discharge carbonization, eventually leading to the through-breakdown under high electric fields. Increasing the creepage distance and improving the fiber groove structure can alleviate the interface degradation issue [19]. Although existing research has advanced our understanding of the failure mechanisms and improvement measures for optical fiber interfaces, the key issue of insufficient interface bonding performance in traditional optical fiber composite insulators remains unresolved. Ensuring high interface reliability in the manufacturing of optical fiber composite insulators remains a significant challenge in the development of modern power grids.

**Figure 1:**
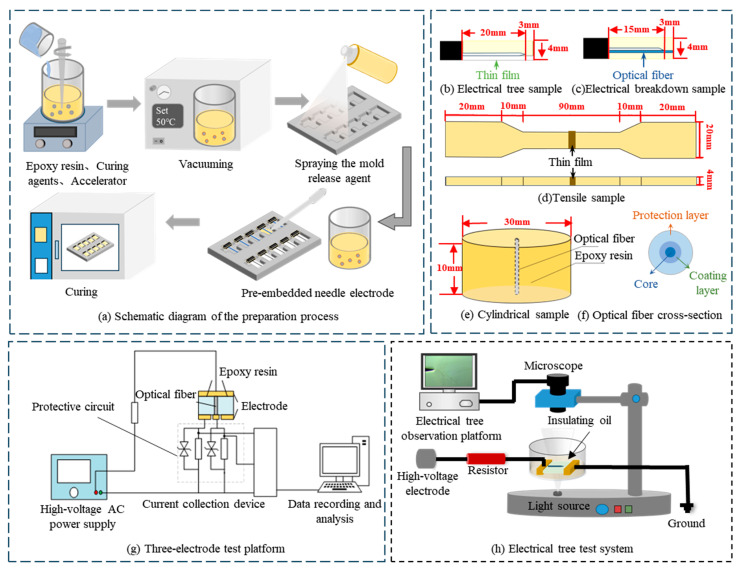
Sample preparation process and testing platform.

In recent years, cycloaliphatic-like epoxy resins (CEP), including cycloaliphatic epoxy resins and hydrogenated bisphenol A epoxy resins, have gained widespread attention in outdoor insulation due to their excellent weather resistance [20], outstanding water resistance, and interface properties [21,22,23,24,25,26]. At the same thickness, the water permeability rate of CEP at room temperature is only 5% that of silicone rubber, significantly limiting the diffusion of water molecules through the sheath to the interface and thus mitigating interface aging and failure [18]. Long-term hygrothermal aging tests show that the leakage current at the silicone rubber and core rod interface increases significantly after 250 h of aging, reaching a maximum of 0.7 mA. Conversely, the leakage current at the CEP and core rod interface remains below 0.1 mA throughout 700 h of aging, maintaining a low level and demonstrating excellent interface aging resistance. Under a strong electric field of 4 kV/cm, the temperature rise at the CEP and core rod interface is significantly lower than that at the silicone rubber and core rod interface [27]. Nie et al. proposed and validated an “S”-type model describing interface aging behavior. The results indicate that, compared to silicone rubber, the interface between CEP and the core rod exhibits a lower interface defect growth index and enhanced resistance to aging [28]. It is evident that the long-term interface stability between CEP and the core rod is excellent. Hence, replacing silicone rubber with CEP as the optical fiber composite insulator sheath material and placing the optical fiber between the CEP and core rod interface is expected to fundamentally address the potential failure risk of traditional optical fiber composite insulator interfaces. However, to protect the optical fiber core, commercial optical fibers are typically coated with protective materials of various compositions (as shown in Figure 1f), and the interface compatibility between these materials and CEP has not been systematically studied. Property differences between the fiber outer materials may still cause significant variations in interface performance with CEP, potentially affecting the overall reliability of the optical fiber composite insulator [29].

Therefore, unlike previous studies that mainly addressed interface aging or macroscopic structural optimization of silicone rubber based optical fiber composite insulators, this work firstly investigates the interfacial compatibility between CEP and optical fibers. Three representative optical fibers with different outer coatings were selected as model systems to systematically investigates how their surface physicochemical properties influence interfacial bonding and insulation performance. For this purpose, multi-scale research methods, such as static contact angle dye penetration, interface insulation characteristics, bonding strength, and molecular simulation, were used. The results establish a quantitative link between interfacial chemistry and insulation reliability and propose a material selection strategy to fundamentally enhance the long-term stability of optical fiber composite insulators.

## 2. Experimental Methods and Molecular Modeling

### 2.1. Sample Preparation

Hydrogenated bisphenol A epoxy resin (HEP, epoxy equivalent weight of 200–230 g/mol), supplied by Beijing Keruite Science and Technology Co., Ltd. (Beijing, China), was selected for CEP. Methyl hexahydrophthalic anhydride (MeHHPA) was used as the curing agent, and *N*,*N*-dimethylbenzylamine (BDMA) as the accelerator, both supplied by Puyang Huicheng Electronic Materials Co., Ltd. (Puyang, China). Three types of optical fibers were provided by Beijing Keruite Science and Technology Co., Ltd. As illustrated in Figure 1f, an optical fiber generally comprises a silica core, an epoxy acrylate (EA) resin coating layer, and an outer protection layer. Among the three fiber types, two are commonly used in optical fiber composite insulators. They have identical silica cores and EA coating layers but differ in their outer protection layers, which consist of ethylene-tetrafluoroethylene copolymer (ETFE) and thermoplastic polyester elastomer (TPEE), respectively. In addition, recent manufacturing efforts have attempted to remove the outer protection layer and embed fibers with only the EA coating into composite insulators, aiming to reduce the number of interfaces and potentially enhance interfacial performance. Therefore, a third fiber type, consisting of a silica core and an EA coating without an outer protection layer, was also selected for comparison. For clarity, these fibers are hereafter referred to as ETFE fiber, TPEE fiber, and EA fiber, respectively. ETFE and TPEE films were sourced from Huayi Plastic Materials and Feilin Laboratory Equipment, respectively. EA film was prepared via the photopolymerization of epoxy acrylate resin.

HEP, MeHHPA, and BDMA were mixed in a beaker in a mass ratio of 100:78.2:1 and stirred continuously until the mixture was homogeneous. The mixture was then placed in a vacuum drying oven at 50 °C for degassing, with each cycle lasting 10 min. This process was repeated three times, with air being evacuated between each cycle. The degassed mixture was then injected into a custom mold, and the curing process was carried out at 100 °C for 2 h, 120 °C for 1 h, and 150 °C for 2 h. After curing, the sample was allowed to cool naturally in the oven to obtain the final product. The sample preparation process is shown in Figure 1a.

Electrical tree growth characteristics effectively reflect the interfacial bonding strength and insulation performance. However, the outer layers of ETFE and TPEE optical fibers are opaque. Although the EA fiber is transparent, electrical trees typically do not form along the fiber–epoxy interface in the early stages, while phenomena such as electrical trees encircling or penetrating the fiber occur in the later stages, as shown in Figure S1 in the Supporting Information (SI). These factors make it challenging to observe the growth of electrical trees clearly. Therefore, thin films made of the same material as the optical fiber’s outer coating were employed to construct an “equivalent interface” for the electrical treeing experiments, as illustrated in Figure 1b. Prior to pouring the epoxy resin mixture, the thin films were laid flat, and a stainless-steel needle electrode was positioned above the film surface. For interfacial breakdown strength tests, samples with a configuration similar to that used in the electrical treeing experiments were employed, in which the metal needle electrode was placed parallel to the optical fiber, as illustrated in Figure 1c. Due to the relatively large diameter of the optical fiber, direct interfacial bonding strength tests, such as the microdrop debonding method, cannot be applied. Accordingly, a tensile testing method based on the “equivalent interface” is developed, as illustrated in Figure 1d. The samples used for dye penetration and water diffusion leakage current tests are shown in Figure 1e.

For clarity, the epoxy samples embedded with ETFE, TPEE, and EA optical fibers are denoted as ETFE–EP, TPEE–EP, and EA–EP, respectively, and were used for dye penetration, interfacial breakdown, and water diffusion leakage current tests. Similarly, the epoxy samples embedded with ETFE, TPEE, and EA films are denoted as f–ETFE–EP, f–TPEE–EP, and f–EA–EP, respectively, and were used for interfacial electrical tree and tensile tests.

### 2.2. Experimental Characterization

#### 2.2.1. Static Contact Angle Measurement

The wettability and surface energy of the optical fiber outer coatings are crucial factors determining its interface bonding strength with epoxy resin. Static contact angle tests were conducted on ETFE, TPEE, and EA fibers, as well as three types of films. Prior to testing, the fibers were cleaned with ethanol to remove contaminants and grease and then fixed in a clamp to ensure the surface was flat and free of curvature. Similarly, the films were also cleaned with ethanol before testing and placed flat on the test bench. Furthermore, the surface energy of different materials was also calculated based on the static contact angle, and the differences were compared. The solid surface energy consists of polar and dispersive components, with the dispersive component approximated as the van der Waals component. By measuring the static contact angles of polar (deionized water) and non-polar (diiodomethane) reference liquids on the material surface [30,31,32,33,34], the surface energy and its components can be obtained using the geometric mean model [35]. The geometric mean model is shown in Equations (1) and (2). The work of adhesion is also an effective parameter for characterizing solid–liquid interfacial interactions and can be calculated using the Young–Dupré equation, as shown in Equation (3) [36].

(1) $$\cos\theta =\frac{2}{\gamma_{L}}\left[\sqrt{\gamma_{L}^{d}\gamma_{S}^{d}}+\sqrt{\gamma_{L}^{p}\gamma_{S}^{p}}\right]-1$$

(2) $$\gamma_{S}=\gamma_{S}^{d}+\gamma_{S}^{p}$$

(3) $$W_{a}=\gamma_{L}(1+\cos\theta )$$

where *θ* represents the static contact angle, and *γ*_L_ and *γ*_S_ represent the surface tensions of the liquid and solid, respectively. *γ*^d^_L_ and *γ*^p^_L_ represent the dispersive and polar components of the liquid surface tension, and *γ*^d^_S_ and *γ*^p^_S_ represent the dispersive and polar components of the solid surface tension, respectively. The surface tension of deionized water is 72.80 mN/m, with polar and dispersive components of 51.00 mN/m and 21.80 mN/m, respectively. The surface tension of diiodomethane is 50.76 mN/m, with a polar component of 0.00 mN/m and a dispersive component of 50.76 mN/m [34]. *W*_a_ represents the work of adhesion.

#### 2.2.2. Dye Penetration Measurement

The dye penetration test, based on capillary action, can quickly identify minor defects at the interface. It was conducted in accordance with IEC 61109:2008 [37] Insulators for overhead lines-Composite suspension and tension insulators for a.c. systems with a nominal voltage greater than 1000 V-Definitions, test methods, and acceptance criteria and GB/T 19519-2014 [38] Insulators for overhead lines-Composite suspension and tension insulators for a.c. systems with a nominal voltage greater than 1000 V-Definitions, test methods, and acceptance criteria, in a glass container was filled with steel balls of 1.5 mm in diameter. Then, 1% magenta ethanol solution (1 g of magenta dissolved in 100 g of ethanol) was poured into the container, raising the liquid level from 2 to 3 mm above the surface of the steel balls. A 10 mm sample was then placed on top of the steel balls, with the optical fiber positioned vertically to the bottom of the container. After standing for 15 min, the dye penetration at the optical fiber–epoxy interface was observed.

#### 2.2.3. Interfacial Insulation Properties Measurement

Electrical tree growth and interface breakdown tests were conducted to evaluate the insulation performance of the epoxy–optical fiber interface. The experimental system is shown in Figure 1h. In the electrical tree growth test, a power frequency voltage was applied, with the voltage gradually increased at a constant rate of 250 V/s until reaching 15 kV, after which it was maintained while the electrical tree growth process was recorded. The interface breakdown test setup was identical to the electrical tree growth test setup. A power frequency voltage was applied, with the voltage continuously increased at a rate of 3 kV/s until breakdown occurred. The voltage at the moment of breakdown was recorded as the breakdown voltage.

#### 2.2.4. Water Diffusion Leakage Current Measurement

Two water diffusion leakage current test specimens were prepared for each group, in accordance with IEC 62217:2012 [39] Polymeric HV insulators for indoor and outdoor use-General definitions, test methods and acceptance criteria and GB/T 22079-2019 [40] HV polymeric insulators for indoor and outdoor use-General definitions, test methods, and acceptance criteria. All samples were immersed in deionized water containing 1% NaCl, with the water temperature maintained at 95 °C. Samples were removed at regular intervals, cooled in room-temperature water for 15 min to ambient temperature, and the leakage current was then measured. During the test, a 4 kV power frequency AC voltage was applied across both ends of the sample and maintained for 1 min. The leakage current waveform was recorded at a sampling frequency of 5 kHz.

#### 2.2.5. Tensile Property Measurement

Tensile tests based on the “equivalent interface” structure were conducted to evaluate the interface bonding strength between the optical fiber outer layer material and the epoxy resin. According to ISO 527-1:2019 [41] Plastics-Determination of tensile properties Part 1: General principles and GB/T 2567-2021 [42] Test methods for properties of resin casting body, five tensile samples were prepared for each group. The samples were stretched at a constant rate of 2 mm/min until fracture. The maximum load at fracture and the stress-strain curve for each sample were recorded, and the tensile strength, *T*, was calculated as shown in Equation (4).

(4) T=P/(B×D)

where *P* represents the maximum tensile force (N), *B* represents the width of the sample’s midsection (mm), and *D* represents the thickness of the sample (mm).

### 2.3. Molecular Simulation Details

Molecular dynamics models were constructed to elucidate the underlying mechanisms behind the differences in interface bonding performance between the three optical fibers and epoxy resin. Since the specific formulation and additive ratios of the commercial optical fiber outer layer materials cannot be fully obtained, only the main components of the outer layer material were selected to construct an idealized interface model, neglecting the potential effects of fillers and additives. This approximation may lead to quantitative differences, but it remains representative of the physical mechanisms of interface bonding. This model can be used to compare the effects of different base materials on interface performance and provide theoretical guidance for subsequent material optimization.

**Figure 2:**
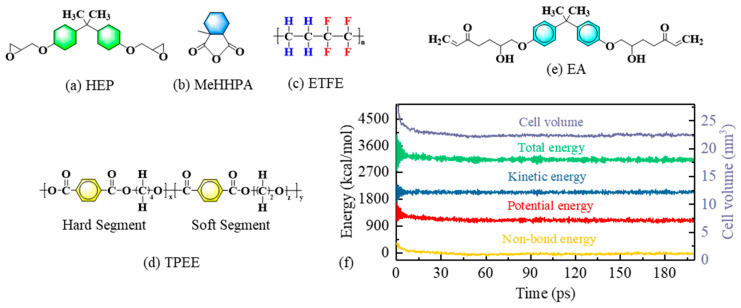
Molecular dynamics models of three fiber–epoxy interfaces: (**a**–**e**) molecular structure, (**f**) physical parameter change curve.

**Figure 3:**
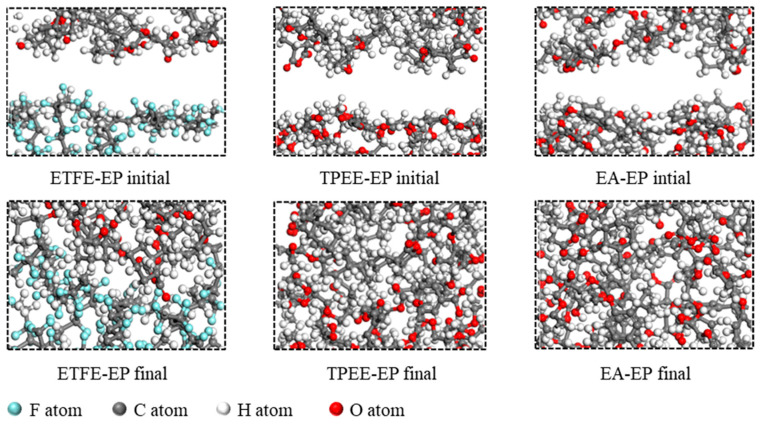
Enlarged view of the interfacial region in the optical fiber–epoxy resin model.

The detailed procedure for constructing the molecular dynamics models is provided in Figure S2 of the Supporting Information. First, the initial molecular structures of HEP, MeHHPA, ETFE, TPEE, and EA were established, as shown in Figure 2a–e. TPEE consists of a hard segment structure of poly-butylene terephthalate (PBT) and a soft segment structure of polyethylene glycol ether (PEG) block copolymer [43,44]. Structural optimization of all initial molecules was performed, using the Smart algorithm. Second, amorphous periodic models of ETFE, TPEE, and cross-linked EA (representing the optical fiber outer layers) were constructed, along with a periodic model of the HEP/MeHHPA blend as the epoxy resin layer. Dynamic calculations were performed using the COMPASS force field under the NVT ensemble, with internal stresses relaxed and unreasonable contacts eliminated. Third, a bilayer interface model containing both the epoxy resin layer and the optical fiber outer layer material was established (as shown in Figure 3), and dynamic calculations were performed under the NPT ensemble with a pressure of 0.1 MPa and a temperature of 300 K until physical parameters stabilized, such as the system energy and cell volume, as shown in Figure 2f. The complete molecular models are provided in Figure S3 of the Supporting Information. The interactions between the epoxy resin and the optical fiber outer layer material at the interface during the relaxation process were analyzed. The Nose method was used for temperature control, and the Berendsen barostat method was used for pressure control.

To further clarify the structure–property relationship between the optical fiber outer layer material and its interface performance, the B3LYP-D3 method of density functional theory was used to calculate the electrostatic potential of different outer layer materials at the 6-31G* level [45], and the results were analyzed using Multiwfn (version 3.8) [46]. Since the molecular structures of ETFE, TPEE, and EA have high polymerization degrees, only the repeating units of these molecules were calculated to reduce computational costs.

## 3. Experimental Results and Discussion

### 3.1. Static Contact Angle

**Figure 4:**
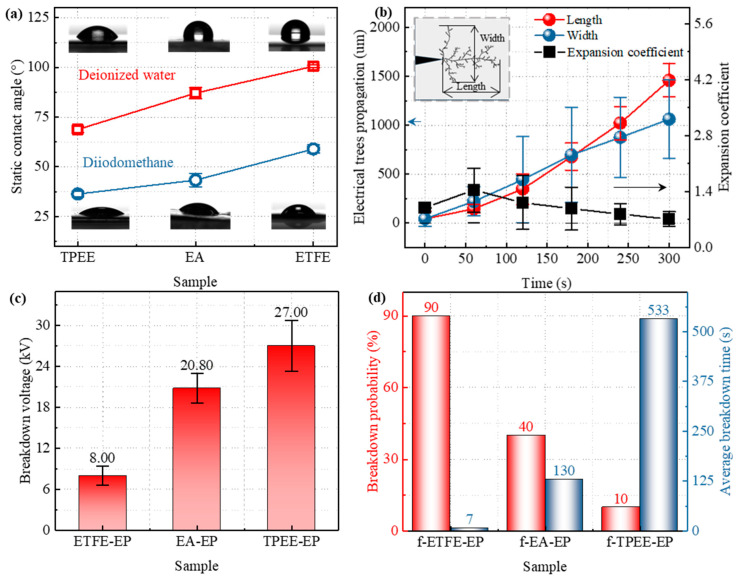
(**a**) Static contact angle; (**b**) electric tree length, width, and expansion coefficient as a function of time; (**c**) breakdown voltage; (**d**) probability of breakdown and average breakdown time within 10 min for the f–TPEE–EP, f–EA–EP, and f–ETFE–EP samples.

The static contact angles of the three optical fibers are presented in Table S1 of the Supporting Information. Since fiber diameter affects contact angle measurement results [47], the smaller radius of the EA fiber limits droplet spreading, leading to an overestimated contact angle. The ETFE and TPEE fibers have the same diameter, but the static contact angle of ETFE is higher than that of TPEE, indicating that the TPEE fiber surface exhibits stronger wettability. The inconsistency in fiber diameters prevents a direct comparison of the static contact angles. Therefore, static contact angle tests are conducted on films made of the same material, as shown in Figure 4a. More detailed contact angle data are provided in Table S2 of the Supporting Information. The static contact angle of the ETFE film with deionized water exceeds 90°, while the static contact angle with diiodomethane is approximately 60°. Both reference liquids exhibit the maximum static contact angle, indicating that the ETFE surface is hydrophobic with poor wettability. In contrast, the TPEE film shows the lowest contact angles with both reference liquids, suggesting a hydrophilic surface with excellent interfacial affinity. The static contact angle of the EA film is intermediate, indicating that its surface affinity lies between that of TPEE and ETFE.

Table 1 presents the surface energy and its components for the three films. It can be seen that the surface energy of the ETFE film is the lowest, at only 29.59 mN/m, while that of the TPEE film is the highest, reaching 48.89 mN/m. The EA film exhibits an intermediate value of 39.68 mN/m, which is consistent with the trend observed in the static contact angle. Similarly, the surface energy of all three films is mainly composed of dispersive components, with polar components being secondary. Among them, TPEE exhibits the largest polar component, 7.49 mN/m, followed by EA at 1.78 mN/m. The polar component of ETFE is the smallest, at only 0.40 mN/m. Since epoxy resin has strong polarity and TPEE has the highest surface energy and the largest polar component, epoxy resin is more likely to spread on the surface of TPEE fibers and form a tightly bonded interface. The low surface energy and minimal polarity of ETFE hinder epoxy spreading, leading to the formation of interfacial air gaps or weak bonding regions, which may act as potential failure sites. In addition, Table 1 indicates that TPEE has the highest work of adhesion, ETFE the lowest, and EA lies in between. This trend also confirms that liquids wet and spread more effectively on TPEE than on ETFE or EA, thereby promoting the formation of a more stable solid–liquid interface [48].

### 3.2. Dye Penetration

**Figure 5:**
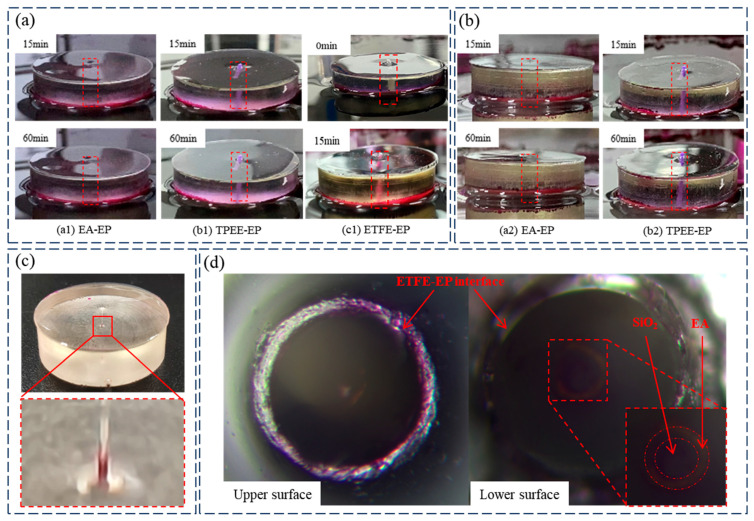
Dye penetration results of three fiber–epoxy specimens: (**a**) at room temperature, (**b**) after thermal cycles, (**c**) EA–EP specimens after thermal cycles, and (**d**) ETFE–EP specimen at room temperature (the position of the optical fiber is indicated by a red dotted line in (**a**,**b**)).

Figure 5a presents the dye penetration results for the ETFE–EP, TPEE–EP, and EA–EP samples. No penetration was observed in the TPEE–EP and EA–EP samples at either 15 or 60 min. However, the ETFE–EP sample exhibited pronounced dye penetration within 15 min, primarily occurring at the interface between the ETFE fiber and the epoxy resin, as illustrated in Figure 5d. These results indicate that strong interfacial bonding is formed between the TPEE and EA fibers and the epoxy matrix, whereas the ETFE fiber fails to establish a tight interface with the epoxy, resulting in the formation of penetrative micro-defects.

Furthermore, TPEE–EP and EA–EP samples were subjected to high–low temperature thermal cycling tests to evaluate the effect of temperature changes on interfacial performance. The thermal cycling involved alternating between 60 °C and −30 °C, with each temperature maintained for 1 h before rapidly switching to the next. After four complete cycles, dye penetration tests were conducted. Figure 5b presents the dye penetration results following thermal cycling. Although no complete dye penetration was observed at the fiber–epoxy interface in either sample after 60 min, partial dye penetration was detected at the bottom of the EA–EP sample, where localized regions of the fiber–epoxy interface exhibited visible dye ingress, as shown in Figure 5c. These results suggest that the interface between the EA fiber and epoxy resin gradually undergoes adhesive degradation under thermal cycling, resulting in the formation of micro-defects. In contrast, no dye penetration was observed at the TPEE–epoxy interface, indicating superior interfacial bonding stability of the TPEE–EP sample under thermal cycling conditions.

### 3.3. Interfacial Insulation Properties

#### 3.3.1. Interfacial Electrical Tree Growth Characteristics

**Figure 6:**
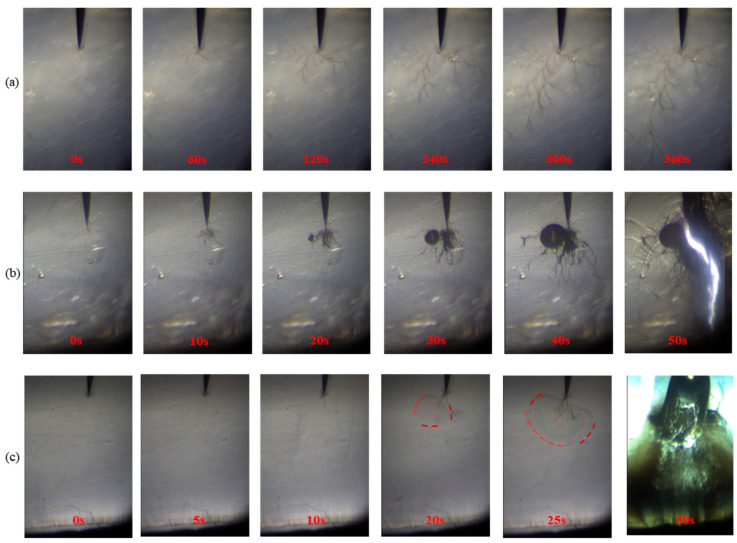
Interfacial electrical tree growth under AC voltage: (**a**) f–TPEE–EP interface, (**b**) f–EA–EP interface, (**c**) f–ETFE–EP interface (the boundary of the local delamination is indicated by a red dotted line).

Figure 6 illustrates the interfacial electrical tree growth characteristics for the f–TPEE–EP, f–ETFE–EP, and f–EA–EP samples. In Figure 6a, the initial state of the f–TPEE–EP sample interface is shown at 0 s, with the high-voltage needle electrode clearly visible. At 60 s, the electrical tree was initiated at the needle tip and rapidly propagated along the direction of the applied electric field. During this process, electrical erosion and thermal decomposition occurred within the tree channel, causing progressive extension along the interface and widening of the main trunk. With increasing duration of the applied voltage, the channel gradually darkened in color, the branching density increased, and numerous filamentary secondary branches developed around the main trunk. Intense partial discharge and oxidation lead to carbonization on the surface of the electrical tree channel, causing its color to darken. After 300 s, the tree growth rate decreased, and by 360 s, the tree had extended close to the breakdown point.

Interface electrical tree growth characteristics of the f–EA–EP sample are slightly different from those of f–TPEE–EP, as shown in Figure 6b. After applying an AC voltage, the electrical tree grows rapidly along the interface toward the ground electrode, taking a branched shape. At 30 s, the shape of the electrical tree transitions from branched to forest-like. As the duration of the applied voltage increases, the electrical tree exhibits rapid growth again, with branched tree growing from the end of the original forest-like structure, developing much faster than the tree propagation speed in the f–EP–TPEE interface. At 50 s, the f–EA–EP interface breaks down, forming a clear conductive channel. Figure 6c presents the morphological evolution of electrical tree at the interface of the f–ETFE–EP sample. Within the first 10 s, the electrical tree grows along the electric field direction. At 20 s, local delamination was observed at the needle tip–interface region. By 25 s, the delaminated area expanded significantly, and the interface undergoes breakdown at 30 s. A comparison reveals that the interface insulation performance of f–EA–EP is weaker than that of f–TPEE–EP, while the electrical tree development at the f–ETFE–EP interface occurs the fastest from initiation to breakdown, indicating that the interface insulation performance between ETFE and epoxy resin is the weakest among the three.

The expansion coefficient is defined as the ratio of the electrical tree width (*W*) to its length (*L*), which quantitatively describes the directional expansion behavior of the interface electrical tree, as shown in Figure 4b. Since electrical tree at the f–ETFE–EP and f–EA–EP interfaces rapidly develops to breakdown, only the electrical tree growth characteristics at the f–TPEE–EP interface are analyzed here. A 15 kV AC voltage is applied at 0 s, with the electrical tree width slightly greater than its length in the early stages. As the voltage application time increases, the growth rate of the electrical tree length increases, making the length greater than the width. The expansion coefficient gradually decreases and then stabilizes at around 0.6, indicating that electrical treeing preferentially propagates along the direction of the applied electric field at later stages.

Under a constant voltage, interfacial breakdown occurs when the electrical tree propagates to the ground electrode. The probability of electrical tree growth to breakdown and the average breakdown time within 10 min under constant voltage of 15 kV for the three interfaces were statistically analyzed as shown in Figure 4d. Among them, only a few samples of the f–TPEE–EP interface exhibited breakdown, with a breakdown probability of just 10%, and the average breakdown time exceeding 500 s. For the f–EA–EP interface, the breakdown probability increased to 40%, while the average breakdown time decreased to 130 s, indicating inferior interfacial insulation performance compared to the f–TPEE–EP sample. The f–ETFE–EP interface is the most prone to breakdown, with a breakdown probability of up to 90% and an average breakdown time of only 7 s. These results demonstrate that the interface between TPEE and epoxy exhibits excellent resistance to electrical tree propagation, providing the best interfacial insulation performance among the three. In contrast, the interface between ETFE and epoxy shows the poorest insulation performance, while the interface between EA and epoxy displays intermediate behavior.

#### 3.3.2. Interfacial Breakdown Characteristics

Interfacial breakdown strength under rapid voltage ramping is also a critical parameter for evaluating interfacial insulation performance. The interfacial electrical treeing tests were conducted using “equivalent interface” samples, whereas the rapid voltage ramping tests were performed on epoxy resin samples embedded with optical fibers, as shown in Figure 1c. The breakdown voltages of the ETFE–EP, TPEE–EP, and EA–EP interfaces under rapid voltage ramping are presented in Figure 4c. Similarly, the average interfacial breakdown voltage of the ETFE–EP sample is the lowest, at only 8 kV. In comparison, the average breakdown voltages of the TPEE–EP and EA–EP samples are 27 kV and 21 kV, respectively, indicating significantly improved interfacial insulation strength. Among them, the TPEE–EP sample exhibits the highest interfacial insulation performance. The variation trend of interfacial breakdown voltage observed in the epoxy resin samples embedded with actual optical fibers is consistent with the electrical treeing behavior obtained from the equivalent interface samples, further confirming the rationality of the equivalent interface design. Moreover, the variation trend in interfacial insulation performance also shows clear consistency with that observed in static contact angle measurements, surface energy values, and dye penetration behavior.

### 3.4. Water Diffusion Leakage Current

**Figure 7:**
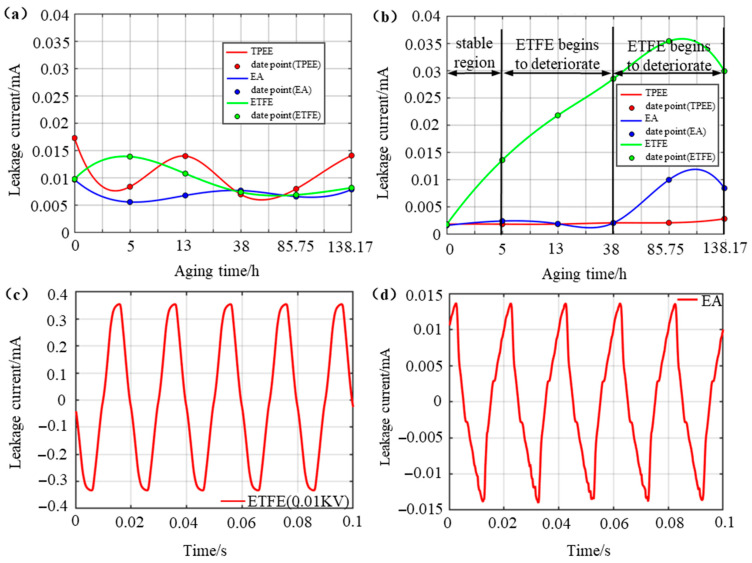
Leakage current of (**a**) the epoxy component, (**b**) the interface component, (**c**) severely degraded ETFE–EP interface at 85 h, and (**d**) interface of EA–EP at 138 h.

Based on the three-electrode test platform, the total leakage current of the ETFE-EP, TPEE-EP, and EA-EP samples under hygrothermal aging were measured, along with the leakage current components at the epoxy resin and interface, as shown in Figure 7a,b. It can be observed that the leakage current through the epoxy resin remained relatively stable for all three samples, indicating that the epoxy resin does not undergo insulation degradation during aging. In contrast, the interfacial leakage current components exhibited significant variation, further confirming that the fiber–epoxy interface is the weak point in insulation reliability. Specifically, during the first 40 h, both the EA–EP and TPEE–EP samples maintain stable interfacial leakage currents without degradation, while the ETFE–EP sample shows a significant increase. At 13 h, the interfacial leakage current of the ETFE–EP sample reached a value ten times higher than that before aging. After 85 h, the TPEE–EP sample still exhibited stable interfacial leakage current without a notable increase. In comparison, the EA–EP sample showed a slight upward trend, while the ETFE–EP sample continued to show a steep rise. Notably, one of the two ETFE–EP samples exhibited an effective leakage current of 0.243 mA under an applied voltage of just 0.01 kV, indicating severe interfacial degradation, as shown in Figure 7c. From 85 h to 138 h, the TPEE–EP sample maintained excellent interfacial stability with no significant increase in leakage current, while the interfacial leakage current of the EA–EP sample continues to rise, though the rate of increase slowed down. At 138 h, the effective leakage current of the EA–EP sample increases to 0.0085 mA, corresponding to 5.25 times that of the original sample, as shown in Figure 7d. For the ETFE–EP sample, the leakage current remained at approximately 16 times the initial value, indicating sustained and severe interfacial degradation. These results demonstrate that the TPEE–EP interface exhibits the highest resistance to hygrothermal aging, while the ETFE–EP interface is the most vulnerable under these conditions. In addition, the water diffusion leakage current of commercial silicone rubber optical fiber composite insulators was also analyzed, as shown in Figure S4 of the Supporting Information. Compared with the commercial optical fiber composite insulators, the TPEE EP interface exhibits a significant performance advantage.

### 3.5. Tensile Property

**Figure 8:**
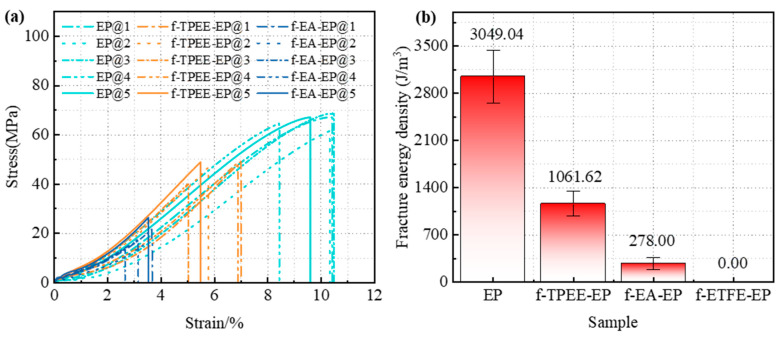
Optical fiber–epoxy mechanical tensile test results: (**a**) stress–strain curves; (**b**) fracture energy density.

During the sample preparation process, it was found that the f–ETFE–EP sample naturally separated from the epoxy resin interface during demolding, making it impossible to prepare a complete dumbbell-shaped tensile sample, as shown in Figure S5c of Supporting Information, indicating extremely poor interface bonding performance between ETFE and epoxy resin. Therefore, only the tensile performance of f–EA–EP, f–TPEE–EP, and pure epoxy resin samples was compared. It can be seen from the Figure 8a that f–EA–EP sample exhibits the average tensile strength of 19.02 MPa and elongation at break of 3.3%. The f–TPEE–EP sample shows significantly higher averages of 48.58 MPa and 6.02%, respectively. The tensile strength and elongation at break of the f–TPEE–EP sample are, respectively, 2.56 times and 1.82 times those of the f–EA–EP sample. The average tensile strength of pure epoxy resin is 65.73 MPa, with an average elongation at break of 9.85%. Compared to pure epoxy resin, the f–TPEE–EP sample retains superior mechanical properties with only 26.09% strength reduction and 38.88% elongation at break decrease. Conversely, the f–EA–EP sample shows substantially greater reductions of 71.06% in tensile strength and 66.50% in elongation at break. Evidently, the interfaces of three films serve as weak points that compromise the mechanical properties of the epoxy matrix. However, the TPEE film has the least negative impact on the tensile performance of epoxy resin, demonstrating significantly superior interfacial bonding compared to both EA and ETFE films.

Figure 8b displays the average fracture energy density at the interfaces. Since the f–ETFE–EP sample fractured during demolding, its fracture energy density is considered to be 0. Among the samples, the f–TPEE–EP sample exhibits highest interface fracture energy density, reaching 1061.62 J/m^3^, approximately four times that of the f–EA–EP sample (278 J/m^3^), further proving that the interface bonding performance between TPEE and epoxy resin is the best, followed by f–EA–EP, with f–ETFE–EP exhibiting the poorest interface bonding performance. This trend is consistent with the results of static contact angle, dye penetration, interface insulation characteristics, and water diffusion leakage current experiments.

## 4. Mechanisms of Interfacial Performance Discrepancies

### 4.1. Interface Bonding Energy

The above experimental results further supports that the surface physicochemical properties of fiber coatings play a critical role in determining interfacial reliability. To further elucidate the structure–property relationship between the molecular structure of optical fiber outer layer materials and their interfacial bonding performance, molecular simulation was constructed for the interfaces between epoxy resin and the three types of fiber coatings. Interface bonding energy is a crucial indicator reflecting the interfacial interaction strength between different materials. The definition of interface bonding energy is given in Equation (5).

(5) $$E_{\mathrm{inter}}=-(E_{\mathrm{all}}-E_{1}-E_{2})$$

where *E*_inter_ represents the interface bonding energy between molecular layers 1 and 2. *E*_all_, *E*_1_, and *E*_2_, respectively, represent the total energy of the system, the total energy of molecular layer 1, and the total energy of molecular layer 2.

**Figure 9:**
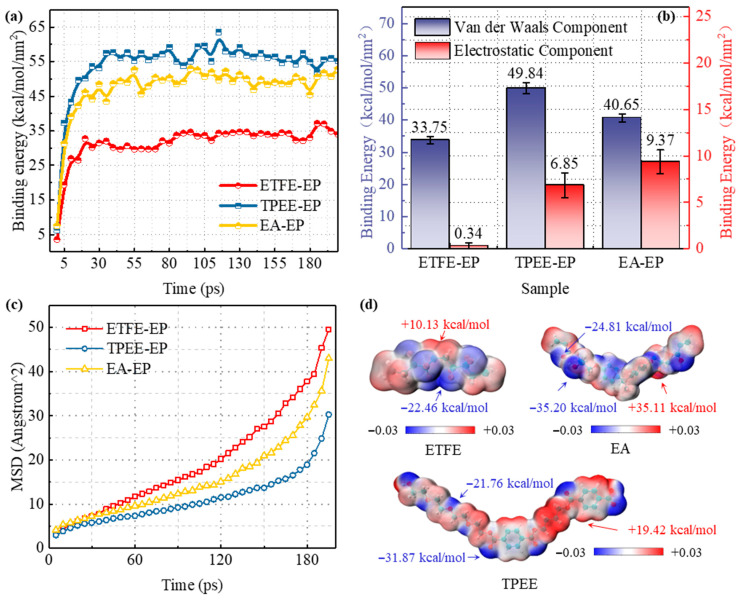
(**a**) Interface binding energy, (**b**) interface bonding energy components, (**c**) mean square displacement, and (**d**) electrostatic potential distribution for the three interface models.

Figure 3 shows the initial and steady-state interface structures of the ETFE–EP, TPEE–EP, and EA–EP models. In the initial state, noticeable free volumes and micro-gaps are presented at the interfaces between the epoxy resin and the outer materials of optical fibers. As the simulation time progresses, the epoxy resin gradually migrates toward the fiber surface, ultimately forming a denser interfacial structure. The significant reduction in interfacial free volume suggests the presence of spontaneous adsorption between the epoxy resin and the outer materials. Figure 9a quantitatively shows the variation of interface bonding energy over time. The interface bonding energy increases rapidly in the initial stages and then stabilizes. Although all three interfaces demonstrate spontaneous adsorption, significant differences exist in the values of interface bonding energy. Specifically, the ETFE–EP interface exhibits the lowest average binding energy at 34.10 kcal·mol^−1^·nm^−2^, whereas interfaces demonstrate significantly higher values of 56.69 kcal·mol^−1^·nm^−2^ and 50.01 kcal·mol^−1^·nm^−2^, respectively. Among these, the TPEE–EP interface exhibits the highest binding energy, indicating the formation of a more tightly bonded interface with the epoxy resin.

Further analysis is conducted on the components contributing to the interfacial binding energy. Similar to the surface energy analysis in Section 3.1, the interface bonding energy is composed of van der Waals interaction energy (non-polar component) and electrostatic interaction energy (polar component). Considering that the interfacial models of the ETFE–EP, TPEE–EP, and EA–EP reached equilibrium after 100 ps, trajectories after 100 ps were selected for analysis, and the average value was taken as the final result. Figure 9b illustrates the components of the interface bonding energy of ETFE–EP, TPEE–EP, and EA–EP models. It can be observed that van der Waals interactions dominate all three interfaces, accounting for over 80% of the total binding energy, while electrostatic interactions constitute a relatively minor portion. Among them, the ETFE–EP interface exhibits the lowest electrostatic interaction energy, indicating that polar interactions at this interface are essentially negligible. In contrast, the TPEE–EP and EA–EP interfaces show not only substantial van der Waals contributions but also significant electrostatic components, suggesting synergistic effects between non-polar and polar interactions. It is noteworthy that the static contact angle analysis reveals that the polar component of surface energy for TPEE is much higher than that of EA. This result appears to deviate from the molecular simulation. The discrepancy may be attributed to the presence of highly polar copolymer units or additives, such as ester and carboxyl groups in commercial TPEE materials, which are not fully captured in the idealized molecular models but may significantly enhance polar interactions at the interface. Consequently, it can be reasonably inferred that polar interactions are more pronounced in the actual TPEE fiber and epoxy resin interface than predicted by simulations, resulting in a stronger interfacial bonding capability. This inference is highly consistent with experimental observations, including dye penetration, interfacial insulation, and tensile performance.

### 4.2. Mean Square Displacement

Carrier transport processes critically determine insulation performance, with molecular chain segment mobility serving as an important factor influencing this process [49]. Figure 9c shows the mean squared displacement (MSD) of epoxy resin molecular chain segments in the ETFE–EP, TPEE–EP, and EA–EP interface models. The results show that the interface bonding energy significantly affects the mobility of the epoxy resin molecular chain segments. Among them, the TPEE–EP interface exhibits the strongest interfacial interactions, leading to the most pronounced restriction on epoxy chain dynamics and resulting in the lowest MSD. Conversely, ETFE–EP interfaces exhibit the highest MSD value, indicating enhanced molecular mobility and a weaker interfacial constraint effect. According to the hopping conduction model, MSD is positively correlated with carrier mobility [50]. Greater MSD values correspond to stronger mobility of molecular chain segments, generating more local free volume and microscopic voids, which facilitate faster carrier migration at the interface layer [51]. Simultaneously, faster chain segment relaxation and increased free volume lead to a significant increase in the carrier mean free path, thereby facilitating impact ionization [52]. Consequently, in the ETFE–EP interface, where chain segment mobility is higher, carrier transport is faster, the mean free path is extended, and electrical trees are more readily initiated, increasing the likelihood of insulation breakdown. In contrast, at the TPEE–EP interface, the mobility of molecular chain segments is substantially restricted, hindering rapid carrier migration and resulting in lower conductivity and superior insulation performance.

### 4.3. Electrostatic Potential

Figure 9d illustrates the electrostatic potential distribution of the ETFE, TPEE, and EA molecules. The ETFE molecule exhibits a highly symmetrical structure with a relatively uniform surface electrostatic potential. The maximum and minimum electrostatic potentials are +10.13 kcal/mol and −22.46 kcal/mol, respectively. The negative electrostatic potential region is primarily concentrated around the F atoms, while the positive electrostatic potential region is located near the H atoms. Compared with TPEE and EA, ETFE exhibits significantly lower surface polarity and cannot form electrostatic interactions with the highly polar functional groups, such as ether and ester bonds, present in the epoxy resin and curing agent (the electrostatic potential of the epoxy and curing agent is shown in Figure S6 of the Supporting Information). Consequently, only van der Waals forces are present at the ETFE–epoxy interface, leading to the lowest interfacial binding energy. In contrast, the electrostatic potential distribution of the TPEE and EA molecules shows pronounced non-uniformity, with significantly enhanced polarity. In the TPEE molecular structure, the O atom in the ester bond exhibits the strongest negative electrostatic potential, followed by the O atom in the ether bond. For EA molecular structure, the H atom in the –OH group shows a notable positive electrostatic potential. The presence of ester bonds, ether bonds, and hydroxyl groups in TPEE imparts strong polarity and allows the formation of potential hydrogen bonds with the polar groups in the epoxy resin and curing agent, thereby enhancing the interface bonding strength. Static contact angle analysis shows that the actual TPEE fiber has a higher polar component than the molecular simulation results, explaining why it forms a stronger interface with epoxy resin compared to the EA fiber. Furthermore, the significant positive and negative electrostatic potentials on the surfaces of the TPEE and EA molecular structures form electron and hole traps, acting as capture sites for charge carriers through electrostatic interactions [53,54,55]. This effectively limits the transport behavior of interface charge carriers, reduces interface conductivity, and improves interface insulation strength. The above simulation analysis provides an atomistic perspective for understanding the intrinsic mechanisms behind the interface performance differences between optical fiber outer materials and epoxy resin.

## 5. Conclusions

This work systematically compares the interface performance of ETFE, TPEE, and EA optical fiber outer materials with epoxy resin, revealing the influence mechanism of the molecular structure on interfacial behavior. TPEE exhibits the highest surface energy and the largest polar component, which promotes the wetting and spreading of epoxy resin and facilitates the formation of a compact and well-bonded interface. As a result, it demonstrates superior interfacial insulation performance, tensile strength, and resistance to hygrothermal aging. In contrast, ETFE has the lowest surface energy and polar component, resulting in poor wetting of the epoxy resin and micro-defects that induce interface failure. Consequently, it has the lowest interface insulation performance, tensile strength, and aging resistance. The interfacial performance of EA falls between those of TPEE and ETFE. The presence of polar groups, such as ester and ether bonds, in the TPEE structure significantly enhances its molecular electrostatic potential. This not only creates more carrier traps at the interface, suppressing the carrier transport process and lowering the interface conductivity, but also strengthens the electrostatic interactions between TPEE and epoxy resin, thereby enhancing the interface bonding energy. The elevated bonding energy consequently imposes significant constraints on the movement of epoxy resin molecular segments, further inhibiting the transport process of interface carriers. The synergistic effects of these factors collectively improve the insulation performance and bonding strength of the TPEE fiber and epoxy resin interface.

## Figures and Tables

**Table 1:** Surface energy and work of adhesion.

Sample	$\gamma_{S}$ (mN/m)	$\gamma_{S}^{d}$ (mN/m)	$\gamma_{S}^{p}$ (mN/m)	Deionized Water *W*_a_ (mJ/m^2^)	Diiodomethane *W*_a_ (mJ/m^2^)
TPEE	48.89	41.40	7.49	99.17	91.68
EA	39.68	37.90	1.78	76.55	87.71
ETFE	29.59	29.19	0.40	59.46	76.98

## Data Availability

The original contributions presented in this study are included in the article/Supplementary Materials. Further inquiries can be directed to the corresponding authors.

## Supplementary Materials

The following supporting information can be downloaded at: https://www.mdpi.com/article/10.3390/ma18163830/s1, Figure S1: Electrical tree morphology in epoxy resin embedded with EA fiber; Figure S2: The detailed procedure for constructing the molecular dynamics models; Figure S3: Molecular model units; Figure S4: Water diffusion leakage current testing of commercial silicone rubber optical fiber composite insulators; Figure S5: Tensile specimen of the equivalent interface; Figure S6: The electrostatic potential distributions of the epoxy resin and curing agent; Table S1: Static contact angle of the optical fibers; Table S2: Static contact angle of the optical fiber films.
