# Supplementary material for: Understanding the Interfacial Behavior of Cycloaliphatic-like Epoxy Resin with Optical Fibers: Insights from Experiments and Molecular Simulations
Source: Materials (Basel). 2025 Aug 15;18(16):3830. doi: 10.3390/ma18163830 (PMC12387235; doi:10.3390/ma18163830)
Supplement: Supplementary file 1 [file materials-18-03830-s001.zip › materials-3783894-supplementary.pdf]

Supporting Information

Understanding the Interfacial Behavior of Cycloaliphatic-Like Epoxy Resin with Optical Fibers: Insights from Experiments and Molecular Simulations

Jianbing Fu[1,2], Zhifan Lin[3], Junhao Luo[3], Yufan Zheng[3], Yuhao Liu[3*], Bin Cao[1*], Fanghui Yin[1], Liming Wang[1*]

1. *Tsinghua Shenzhen International Graduate School, Tsinghua University, Shenzhen 518055, China*
2. *China Electric Power Equipment and Technology Co., Ltd., Beijing 100032, China*
3. *College of Electrical Engineering and Automation, Fuzhou University, Fuzhou, Fujian 350108, China*

*Corresponding author: Yuhao Liu (yhliu@fzu.edu.cn), Bin Cao (cao.bin@sz.tsinghua.edu.cn) and Liming Wang (wanglm@sz.tsinghua.edu.cn)

S1 Experimental Methods and Molecular Modeling

S1.1 Interfacial electrical tree

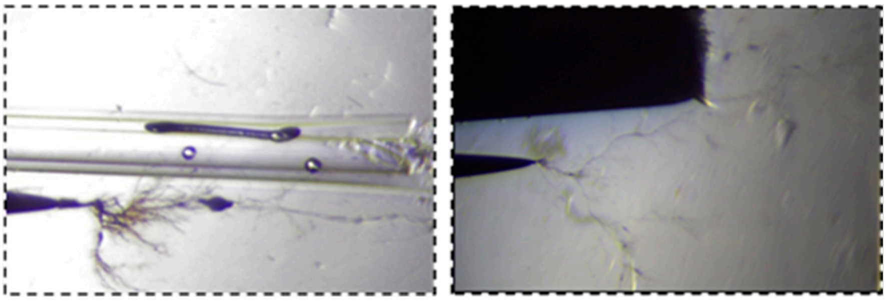

Figure S1. Electrical tree morphology in epoxy resin embedded with EA fiber.

S1.2 Molecular simulation details

① **Initial molecular models**: Molecular models of ETFE, TPEE, EA, the resin HEP, and the curing agent MeHHPA were established based on the Fig. 2(a-e), and all structures were optimized.

② **Amorphous periodic models**: Amorphous periodic models of ETFE, TPEE, and EA were constructed to represent the fiber layers, while an amorphous periodic model of the HEP/MeHHPA mixture was built to represent the epoxy resin layer. These amorphous models were equilibrated in the NVT ensemble at 300 K to relax internal stresses.

③ **Interface models and equilibration**: Bilayer interface models combining the fiber layer and the epoxy resin layer were then created. MD simulations were performed in the NPT ensemble at 300 K and 0.1 MPa until the physical parameters (e.g., total energy and cell volume) stabilized. Frames from the equilibrated system were subsequently selected to analyze the interfacial interactions between the fiber layer and the epoxy resin layer.

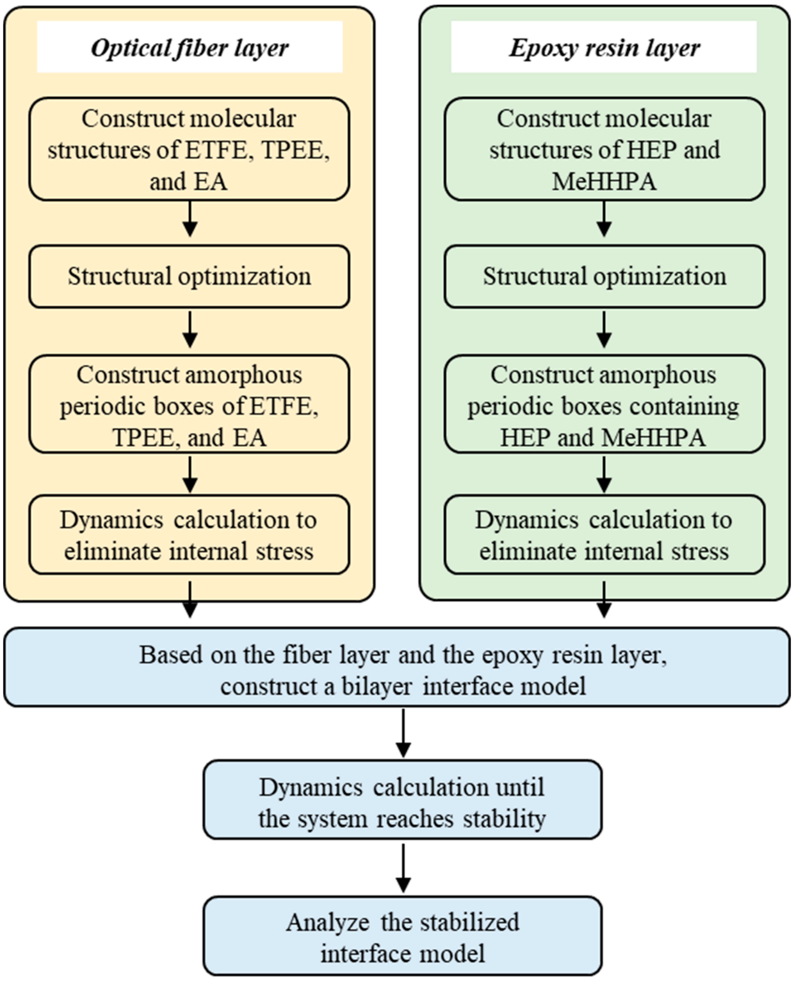

Figure S2. The detailed procedure for constructing the molecular dynamics models.

S1.3 Molecular model units

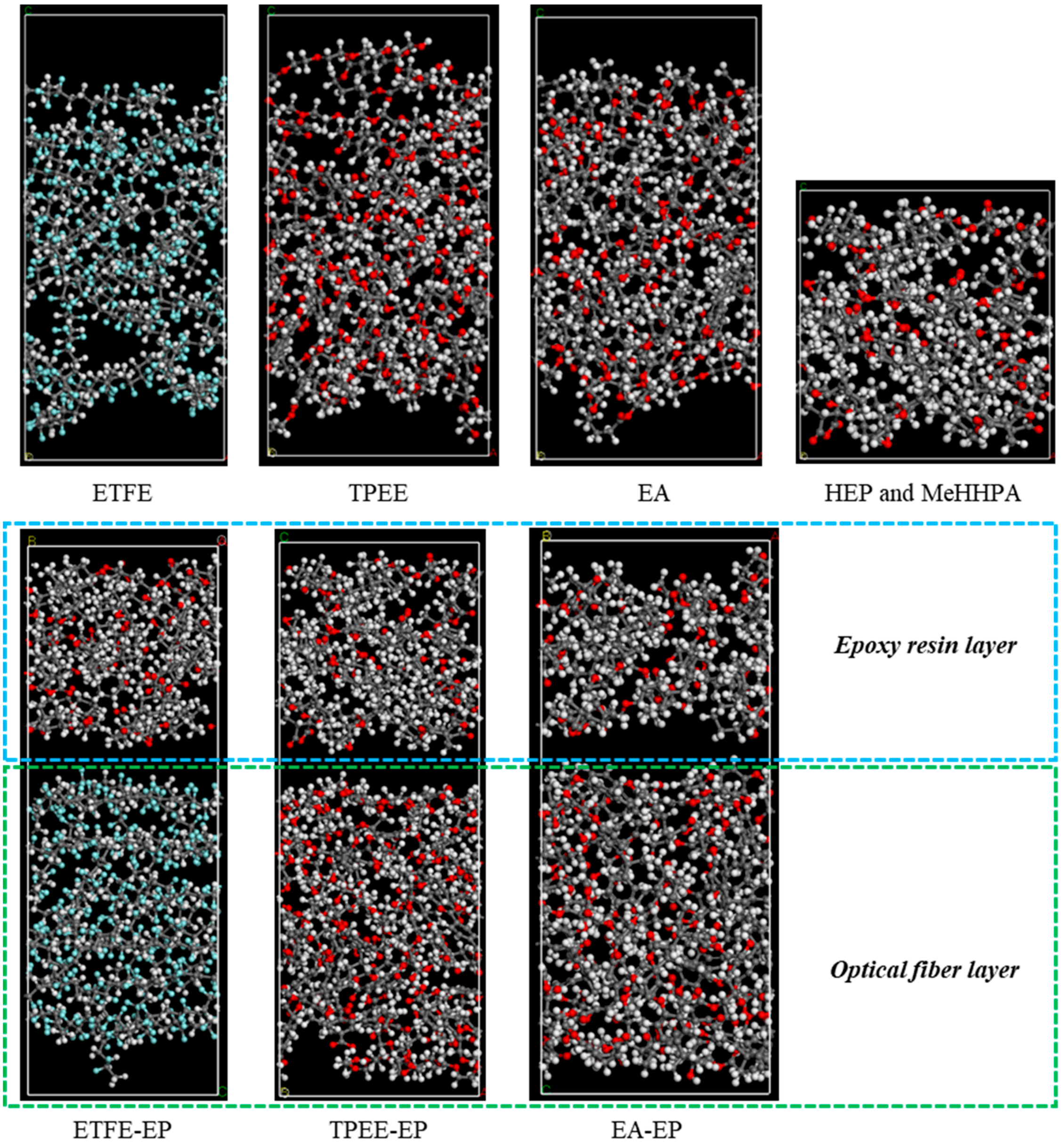

Figure S3. Molecular model units.

S2 Experimental Methods and Molecular Modeling

S2.1 Static contact angle

Table S1 Static contact angle of the optical fibers

Sample	Deionized water	Diiodomethane
TPEE	100.56°	69.78°
EA	97.40°	71.50°
ETFE	30.07°	75.42°

Table S2 Static contact angle of the optical fiber films

Deionized water			Diiodomethane		
EA	TPEE	ETFE	EA	TPEE	ETFE
85.5°	70.3°	100.5°	47.0°	37.7°	55.9°
85.5°	70.2°	100.5°	47.0°	37.7°	55.9°
90.6°	69.8°	100.5°	45.1°	36.1°	58.8°
90.6°	69.8°	100.5°	45.1°	36.1°	58.8°
88.4°	66.7°	99.9°	42.5°	34.2°	59.5°
88.4°	66.7°	99.9°	42.5°	34.2°	59.5°
83.7°	68.3°	101.4°	38.5°	37.1°	61.4°
83.7°	68.3°	101.3°	38.5°	37.1°	61.4°

S2.2 Water diffusion leakage current testing of commercial silicone rubber optical fiber composite insulators

The interfacial performance of a commercial silicone-rubber optical fiber composite insulator was analyzed, as shown in Fig. S4. Initially, the leakage current was low; however, after 100 h of boiling water aging, breakdown occurred at the fiber interface, leading to a significant deterioration of interfacial performance, as shown in Fig. S4(c).

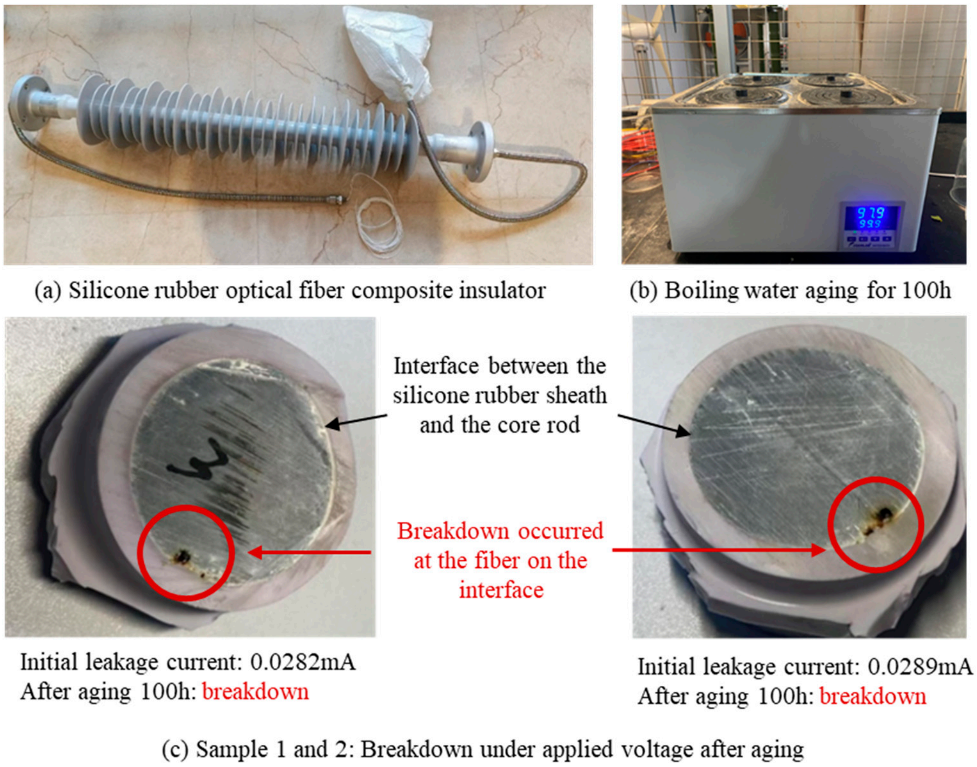

Figure S4. Water diffusion leakage current testing of commercial silicone rubber optical fiber composite insulators.

S2.3 Details of molecular dynamics simulation

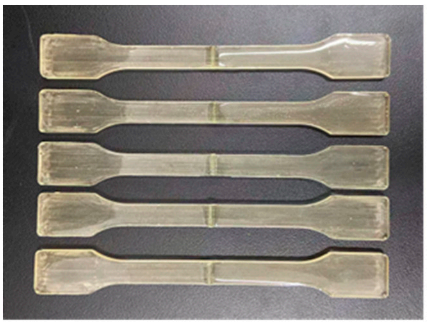

(a) f-EA-EP

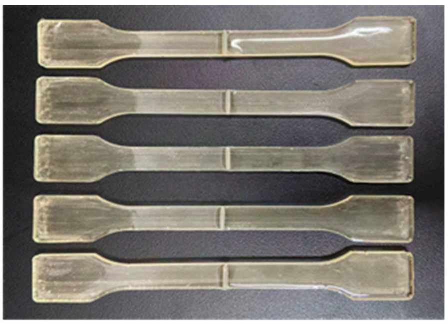

(b) f-TPEE-EP

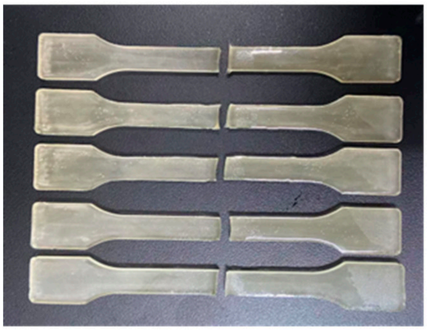

(c) f-ETFE-EP

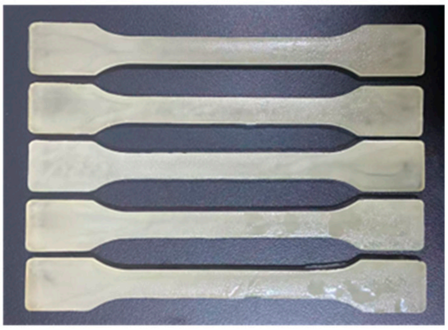

(d) pure epoxy resin

Figure S5. Tensile specimen of the equivalent interface

S3 Mechanisms of interfacial performance discrepancies

S3.1 Electrostatic potential

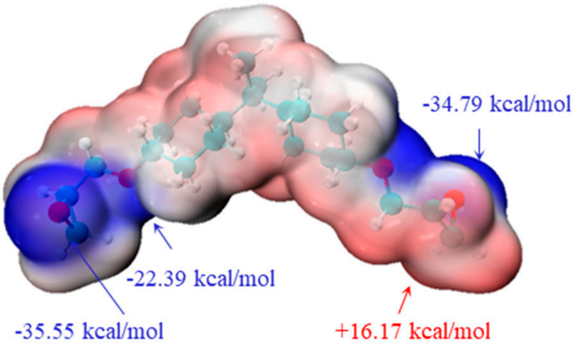

(a) HEP

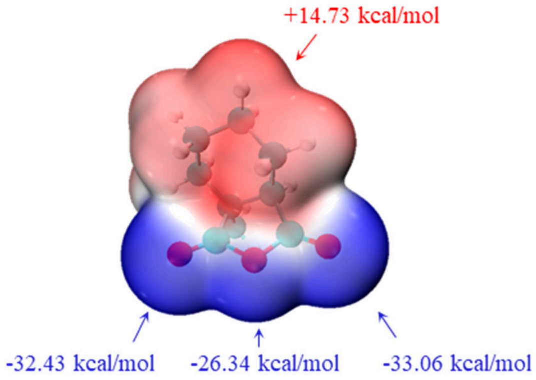

(b) MeHHPA

Figure S6. The electrostatic potential distributions of the epoxy resin and curing agent.
